# Harnessing the power of novel animal-free test methods for the development of COVID-19 drugs and vaccines

**DOI:** 10.1007/s00204-020-02787-2

**Published:** 2020-05-23

**Authors:** Francois Busquet, Thomas Hartung, Giorgia Pallocca, Costanza Rovida, Marcel Leist

**Affiliations:** 1 CAAT-Europe at the University of Konstanz, 78457 Konstanz, Germany; 2 ALTERTOX, 1000 Brussels, Belgium; 3 CAAT, Bloomberg School of Public Health, Johns Hopkins University, Baltimore, MD 21287 USA; 4 In Vitro Toxicology and Biomedicine, Department Inaugurated By the Doerenkamp-Zbinden Foundation, University of Konstanz, 78457 Konstanz, Germany

## Abstract

The COVID-19-inducing virus, SARS-CoV2, is likely to remain a threat to human health unless efficient drugs or vaccines become available. Given the extent of the current pandemic (people in over one hundred countries infected) and its disastrous effect on world economy (associated with limitations of human rights), speedy drug discovery is critical. In this situation, past investments into the development of new (animal-free) approach methods (NAM) for drug safety, efficacy, and quality evaluation can be leveraged. For this, we provide an overview of repurposing ideas to shortcut drug development times. Animal-based testing would be too lengthy, and it largely fails, when a pathogen is species-specific or if the desired drug is based on specific features of human biology. Fortunately, industry has already largely shifted to NAM, and some public funding programs have advanced the development of animal-free technologies. For instance, NAM can predict genotoxicity (a major aspect of carcinogenicity) within days, human antibodies targeting virus epitopes can be generated in molecular biology laboratories within weeks, and various human cell-based organoids are available to test virus infectivity and the biological processes controlling them. The European Medicines Agency (EMA) has formed an expert group to pave the way for the use of such approaches for accelerated drug development. This situation illustrates the importance of diversification in drug discovery strategies and clearly shows the shortcomings of an approach that invests 95% of resources into a single technology (animal experimentation) in the face of challenges that require alternative approaches.

**Electronic supplementary material:**

The online version of this article (10.1007/s00204-020-02787-2) contains supplementary material, which is available to authorized users.

## Introduction

The spread of the COVID-19 pandemic is seriously challenging the scientific community. The quest is not only to find suitable vaccines and/or drugs but to do it as fast as possible. Unlike many other diseases, there is not just a medical need, but also increasing pressure from key economic and political decision-makers. The President of the European Commission (EC), Ursula von der Leyen, for example, voiced hopes that a vaccine would be available by autumn 2020 (Wheaton 2020). In light of these and similar remarks, it is worth looking at the tools and the regulatory mechanisms that may allow us to overcome this unprecedented health crisis. As viral infections are the prototypic species-specific diseases, they make animal testing challenging even without such time pressures. Their duration and costs, especially when genetically modified strains susceptible to the disease need to be bred, do not support such ambitious goals, while modern bioengineered human (multiple) organ models lend themselves to antiviral drug development. Some countries have already started human clinical trials after only minimal safety testing in animals, for example at the National Institutes of Health (NIH) (Roberts 2020; Boodman 2020). The EMA has provided updates on treatments and vaccines under development against COVID-19 in its last briefing (1), with anticipated timelines for market entry not before 2021 and (2) an outline for the way forward to facilitate market access and authorization (EMA 2020a). Exceptional funding efforts were also made available via the public–private partnership of the Innovative Medicines Initiative (IMI) (https://bit.ly/3aDRbUP) for boosting development of therapeutics and diagnostics to tackle current and future coronavirus outbreaks (IMI 2020; EC Research and Innovation 2020).

Here, we will explore how NAM can accelerate such developments.

## Four testing programs for drug and vaccine discovery that may be accelerated by the use of NAM

Drug and vaccine development do not differ in principle but in detail (Meigs et al. 2018). They go through the same steps of pre-clinical and clinical development, acceptance, and post-market surveillance. Vaccine developments tend to be longer (8–18 years vs. 8–12 years for drug trials). The clinical trials are often larger and longer as (risk) populations need to be vaccinated for often rare events. Due to the nature of biologicals, which are often produced by fermenting or types of cell culture, vaccines frequently need batch release controls. As public health measures, vaccines face even higher pricing pressures. All of this makes the development of vaccines less attractive for pharma, and indeed, there are only a few major pharma companies engaging in their development. To support vaccine development, governmental players and nonprofit foundations are co-funding R&D and sometimes production. Interestingly, more than 80% of global vaccine producers are European, while more than 40% of vaccine consumption is in the US (Meigs et al. 2018).

Altogether, drug discovery (R&D) differs from many other scientific disciplines, and understanding its major components helps us appreciate how new tools can be used to accelerate the process. Models, also called test systems, are essential for the R&D process. Traditional models are based on experimental animals. Novel approaches are animal-free and use tissue cultures or computational techniques. A third type of test “model” must not be forgotten, however, as it can complement the others: healthy or diseased humans. The use of such tools in R&D programs need to answer four entirely different questions. Therefore, they are used in four very different testing strategies, with each of the approaches having its own particular models and providing specific opportunities for the use of NAM.

**Fig. 1:**
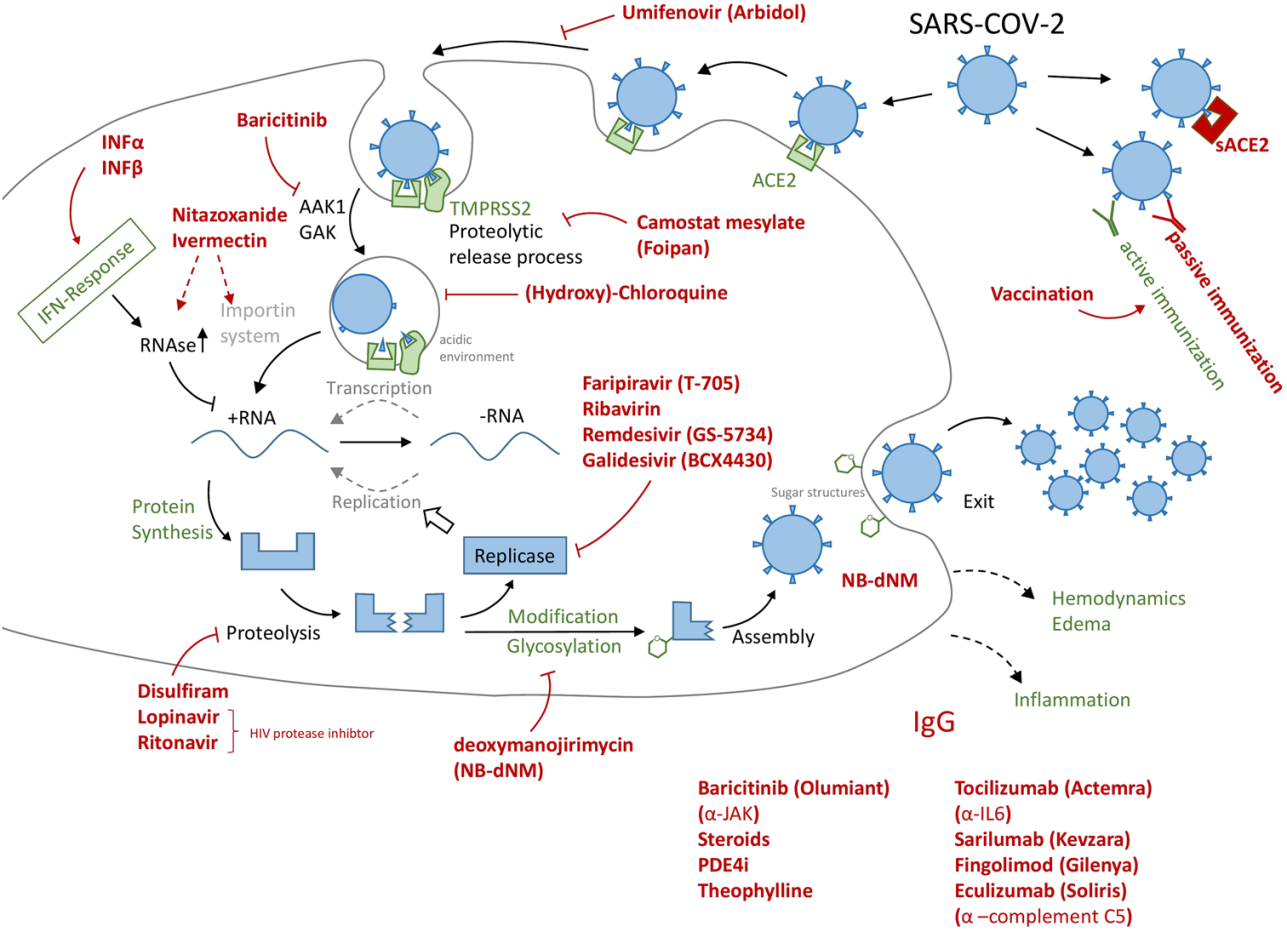
Overview of some drug repurposing approaches for COVID-19. The black structures indicate the target cell, and green structures refer to host components and processes. The virus or its elements are shown in blue, while drugs are presented in red. The drugs in the lower right corner have been considered to fight inflammation or to smoothen a potential cytokine storm, *i.e.* they act on processes not directly linked to the virus or its target cell (type II pneumocyte) in the lung. Arrows refer to drug targets or target processes, as they have been suggested in the literature. Sometimes the exact targets, and respective drug efficacy are not known. The selection of drugs (and drug types) is exemplary, and good other approaches may be missing, while some of those shown may be of little use. The intention was to highlight the broad variety of approaches, many of them also used clinically in case studies, compassionate trials or initial stages of testing programs. At this stage, NAM would be useful to (i) determine the target structures, (ii) define exact biological effects of drug candidates, (iii) investigate and quantify efficacy, and (iv) close gaps in safety knowledge on some candidates not yet approved or used clinically in other settings (e.g. dosing schedule). *ACE2* angiotensin converting enzyme-2, *sACE2* recombinant soluble domain of ACE2, *IFN* interferon, *TMPRSS2* transmembrane serine protease subtype-2, *AAK1, GAK* enzymes involved in endocytosis, *JAK* kinase (janus kinase family) involved in cytokine signaling (inflammation) (color figure online)
- 2. *Safety* An entirely different set of models and tests is used to evaluate whether a drug is well-tolerated and without adverse side-effects (*i.e.*, toxicity). For instance, it needs to be assured that it does not block important functions of the human body or attack important structures within human cells or organs.
- 3. *Quality* Once a safe and efficacious vaccine (or drug) has been found, it needs to be produced at high, and more importantly, constant quality. The testing program differs fundamentally from safety and efficacy, as it needs to be applied to each lot of vaccine (or drug) to control for impurities and contaminants.
- 4. *Target discovery* Last in this list, but first in the R&D process, is a testing program designed to yield useful drug targets. Again, different models and strategies are required to find, for example, the key steps of virus-host cell interaction or to identify processes in human cells that could be used as a target to decrease hyper-inflammation. As some of these targets may be known from other diseases or research, they may provide one of the fastest ways to find suitable drugs: a strategy called *re-purposing.* A typical example may be glycosylation inhibitors (Fig. 1), which were developed for various neurological diseases but may also be considered for viral infections (Park 2019; Parsons et al. 2019; Tortorici et al. 2019), and could be tested quickly with modern NAM via metabolic glycoengineering (Kranaster et al. 2020).

The scopes of the four approaches are:

- 1. *Efficacy* This is what usually comes to mind when we think of drugs (or vaccines). Even if the principles of efficacy and action are clear (e.g., preventing viral replication or blocking virus entry into a cell), and also when the particular molecular target is known (e.g., the viral RNA polymerase or docking receptor) (Fig. 1), finding a drug candidate that interacts with the target, and also fulfills some other features essential to make it a drug (e.g., being taken up by the body and being transported to its target site), is a tedious and highly resource-consuming process.

For all four approaches, COVID-19 research can now benefit from the investments made into development of NAM over the past 20 years. From a European perspective, one of the two key developments has been the establishment of the European Center for the Validation of Alternative Methods (EURL-ECVAM) within the EC own research infrastructure, the Joint Research Center (JRC). This was followed by the creation of a network of European reference laboratories (EU-NETVAL) coordinated by EURL-ECVAM. The second major push for the development of NAM came from substantial funding of this research by the EC, comprising up to 50 M €/year for the last 15 years since the Framework Program 6 (FP6). This also includes the IMI funding. One current project is EU-ToxRisk, which develops animal-free safety testing approaches with a budget of 30 M € (Daneshian et al. 2016; Graepel et al. 2019). Several national funding programs have also contributed substantially to the development of NAM, particularly in The Netherlands, Germany, and the UK. Legislation in the EU, such as the ban of animal testing for cosmetics, has been a strong incentive for industry to launch their own (and very successful) research programs on NAM safety and quality control (Beilmann et al. 2019). In the US, the National Academy of Sciences (NAS) 2007 report on the future of toxicity testing in the twenty-first century (NRC 2007; Leist et al. 2008, 2014; Krewski et al. 2010, 2020) was pivotal, triggering a chain of events culminating with roadmaps from major agencies (EPA, FDA) to reduce and ultimately abandon animal testing (Collins et al. 2008; Tice et al. 2013; Bouhifd et al. 2015; FDA 2017; Thomas et al. 2018; EPA 2019). A second notable report from a committee of the NAS addressed drug efficacy for countermeasures to terrorism (biological weapons) (NRC 2011; Hartung and Zurlo 2012). Here, a clear recommendation was given to focus solely on NAM for reasons of speed and predictivity. In parallel to these highly visible government activities, industry moved from largely animal-based efficacy testing in the 1980s to predominantly (90%) animal-free efficacy research methods today. The same is true for academia and especially for industry-driven research on target discovery (Busquet et al. 2020). Here, cell culture-based methods combined with modern molecular biology and stem cell technology play a major role, but the use of computational research (in silico models) has recently increased as well (Pinero et al. 2018; Vamathevan et al. 2019), in parallel with an increasing awareness of shortcomings of animal models (Olson et al. 2000; Hartung and Leist 2008; Leist and Hartung 2013).

The use of NAM for these four testing programs is detailed below.

## Use of NAM for efficacy testing during drug and vaccine development

### Drug development

What is special about SARS-2-CoV-19 (COVID-19)? First, we have no animal disease model. It seems to be most closely related to coronaviruses in Asian bats and reptiles, but these do not lend themselves as lab animals and do not show (as far as we know) the pathology found in humans. The typical reflex when there is no animal model is to suggest using non-human primates (NHP) or even great apes. This has not helped us with HIV or hepatitis C., as shown by the US National Academy of Sciences (NAS 2011) research with great apes. The other common response is to breed or genetically modify lab animals such as mice to render them susceptible, as was done for the earlier SARS virus (McCray et al. 2007). However, this misses the most obvious opportunity: human microphysiological systems (MPS) that have become available over the last decade due to stem cell technology and bioengineering (Alepee et al. 2014; Marx et al. 2016, 2020; Ewart et al. 2018). We have shown earlier how viral infections can be studied in human BrainSpheres (“mini-brains”) (Abreu et al. 2018) for Zika and Dengue, as well as with others, including SARS-2-COVID-19 (to be published shortly). Others are also doing similar research in various organs, such as the European OrganoVir education network (https://organovir.com). Many groups building on these models will now leverage them for COVID-19 R&D. An important finding based on organoids was published by a research group in Vienna that focused on soluble viral receptors (sACE2) as therapy (Monteil et al. 2020). There are, however, limitations on research done under biosafety level 3 guidelines for this virus, with sophisticated systems of pumps, sensors, microscopes, and difficult-to-sterilize equipment. Specific consideration should thus be given to the design of simple and robust models—typically more so organoids than organ-on-chip systems. For respiratory tissue, commercial offers currently exist from Epithelix and MatTek (Gordon et al. 2015). It seems, however, that the life-threatening course of COVID-19 occurs through a cytokine storm with acute respiratory distress syndrome and multiple organ failures. These forms of septic shock have been especially resistant to drug identification, killing drug companies’ sepsis programs as septic patients in the clinic. It is rather unlikely that the multi-organ failures in COVID-19, once the disease has developed, represent a different situation.

### Drug repurposing

The Cochrane study register for COVID-19 listed (on 19 April 2020) a total of 1684 studies launched, with 637 of them being interventional and 781 aimed at treatment and management (https://covid-19.cochrane.org). This, however, has to be understood proportionally in light of about 55,000 clinical trials listed in 2019 by the World Health Organization (WHO) (https://bit.ly/2y2TzqS). Still, the speed of getting these started is impressive. This shows that drug repurposing is mainly occurring. This should not be surprising, as Drugbank lists 13,543 drugs available (11,388 small molecules, 2155 biologicals), of which 4002 are approved (https://www.drugbank.ca/stats). This rich portfolio, which has typically already undergone some safety testing, allows it to move quickly ahead. Initially, the focus is entirely on NAM. Cellular infectivity can, for instance, be tested on cell cultures (Vero cells or organoids), and molecular viral targets, like the polymerase, are initially tested in recombinant form (biochemical assays). Both methods allow high throughput (thousands of samples) and provide much more robust and exacting data than any animal experiment could deliver. More complex questions concern whether a potential drug would reach its target tissue, and whether it would also act in a complex disease setting (inflammation, altered pH, or in a variety of cell types). Moreover here, combinations of complex in vitro models, and of in silico approaches (physiology-based pharmacokinetic (PBK) modelling) are well established and routinely used in drug industry and academia. Considering that a share of repurposing drug candidates comes from HIV drug research, there is now ample opportunity to test failed or discarded substances as well as supportive therapies. The first results with established AIDS therapies, however, were disappointing (https://bit.ly/3cMmsXe). The WHO summarized the limited evidence so far (https://bit.ly/2S6NIrF) but discussions and hopes continue (Murphy 2020) (Fig. 1).

### The challenge of combination therapies

In the case of HIV, treatments ultimately became successful when combining (originally four, now three) different treatments, known as HAART (highly active antiretroviral therapy). However, our understanding of HIV differs from COVID-19 in two critical aspects: the extent of immune escape and persistence in the body. Still, it might be necessary to combine treatments, which is always a challenge for drug development, approval, and practical patient care. NAM may be used to identify the most effective combination of targets (synergy) and to find the most beneficial combination of drugs (concentration ratios) before such efforts are carried out in patients.

### Vaccine development

Already more than 115 vaccines for COVID-19 are under development (Thanh et al. 2020).

As most organoids and MPS are devoid of an immune component, they lend themselves less as a substitute, though the addition of immune cells is becoming more common. The addition of antibodies or serum of vaccinated animals or recovered humans to these models to test their efficacy, instead of infecting the animals, is an opportunity here. A most promising development is artificial lymph nodes, as reported first by Suematsu and Watanabe (2004). Giese and Marx (2014) summarized the progress in developing and optimizing a 3D human artificial lymph node model to mimic the interface between the innate and adaptive immune response in vitro (Giese et al. 2006; see also Cupedo et al. 2012). Such models have shown merit for the evaluation of the immunogenicity of influenza vaccines (Drake et al. 2012). They certainly represent a prime non-animal method for COVID-19 vaccine development.

While vaccine development usually targets active vaccination, *i.e*., exposure of humans to relevant antigens, followed by an “active” immune response, passive vaccinations can also be envisaged (Fig. 1). In this process, the antibodies against viral antigens are produced by biotechnology methods and then injected. This is best known for emergency vaccines for tetanus. A variant of this approach is the isolation of antibodies from the serum of COVID-19 survivors for use in patients (JHSPH 2020; Casadevall and Pirofski 2020). The traditional (old-fashioned) way of antibody generation uses animal models. Nowadays, monoclonal antibodies can be produced entirely animal-free by molecular biology techniques (e.g., phage display) (Cosson and Hartley 2016; Gray et al. 2016; Almagro et al. 2019; Zuang et al. 2019).

For the use of animals, there are also limitations due to pronounced species-specific differences in pathogenesis with antigen recognition, in immune reactivity of non-lymphoid and lymphoid tissues, and in the systemic orchestration of immunity at the organism level.

The first successful attempt on rhesus macaques was just confirmed on April 19th, 2020 (Gao et al. 2020). However, concerns have been raised about this approach due to the fact that monkeys do not develop the most severe symptoms that SARS-CoV-2 causes in humans (Cohen 2020).

Therefore, a fast transition from initial trials in vitro to the next step in humans is often taken in COVID-19 research.

## Use of NAM for safety testing requirements

### Regulatory vaccine approval

In the US, an IND (Investigational New Drug) application to the FDA (Food and Drug Administration) starts with the clinical phase describing the manufacturing and testing processes, laboratory reports, and the proposed study, as well as approval by the institutional review board at the institution where the clinical trial will be conducted. The FDA has 30 days to approve the application. Clinical studies with human subjects can then start, similar to those in clinical drug development:

- *Phase I* Vaccine Trials (typically 20–80 adults, may be non-blinded) to assess safety, type, and extent of the immune response
- *Phase II* Vaccine Trials (typically several hundred individuals, often groups at risk of acquiring the disease). These trials are randomized and well-controlled and include a placebo group to study safety, immunogenicity, proposed doses, schedule of immunizations, and method of delivery.
- *Phase III* Vaccine Trials (involving thousands to tens of thousands of people). These are randomized and double-blind studies against a placebo to assess safety, rare side effects, efficacy (prevention of disease, prevention of infection with the pathogen or production of antibodies, or other types of immune responses related to the pathogen).
- *Approval and Licensure* Submission of a Biologics License Application to the FDA, then FDA will inspect the factory and approve the labeling of the vaccine.
- *Post-Licensure Monitoring* of Vaccines includes Phase IV trials, the Vaccine Adverse Event Reporting System (voluntary, established by the Centers for Disease Control and Prevention (CDC) and FDA in 1990), and the Vaccine Safety Datalink (a collection of linked databases containing information from large medical groups established by CDC in 1990).

It is difficult to imagine how such a process could be shortened to a year or 18 months, as has been recently suggested by policymakers.

### Safety testing of drugs and vaccines

NAM have been described in thousands of articles in the past. There have been large successes in some toxicological areas, but also gaps to be filled (Basketter et al. 2012; Leist et al. 2014). These approaches have contributed to limit animal use in toxicology, but much less in biomedical research (Daneshian et al. 2015; Busquet et al. 2020). The use of NAM is not just an animal welfare issue. It is an issue of time—or, more precisely, time to market. In a worldwide pandemic, speed of drug development carries enormous weight. A traditional full safety package based on animals includes long-time chronic dosing (up to two years) followed by lengthy histopathology evaluations. However, shortcuts seem possible. To understand applicability for COVID-19, exact testing needs must be reviewed. Depending on the type of use (duration of treatment, application method(s), use in pregnancy, and other susceptible populations), the safety data package for initial human use is rather light. The speed with which more than 500 clinical trials have begun for COVID-19 shows that shortcuts have been taken, and may generate discussions on what might be possible more generally, for common practices after the pandemic (Bruckner 2020). In the case of COVID-19, the dosing time for a drug is very short (often 2 weeks or less), and in many cases pregnancies (i.e., reproductive and developmental toxicity issues) can be excluded. What is needed in this situation? (1) The drug should not be acutely toxic; (2) it should not trigger cancer; and (3) it should not interfere with other drugs.

Concerning (1), the situation for NAM is very favorable. Accepted methods are available for most acute and topical toxicities. Also, acute systemic toxicity may be predicted, possibly in combination with a limited number of animal experiments (Hamm et al. 2017; Strickland et al. 2018).

Concerning (2), one needs to distinguish genotoxic and non-genotoxic carcinogenicity. Genotoxicity is the most successful area for NAM, and has been addressed for decades in drug development. The available methods are very fast (days) and highly sensitive (Corvi and Madia 2017; Corvi et al. 2019). Non-genotoxic carcinogenicity is harder to predict (from animals or NAM), and it shows pronounced species differences (Hernandez et al. 2009). One fundamental fact is that non-genotoxic carcinogens require usually long exposure times. Thus, the short treatment of COVID-19 minimizes this issue. Moreover, NAM have been developed to identify typical mechanisms contributing to non-genotoxic carcinogenicity (e.g., nuclear receptor activation) (Hernandez et al. 2009; Jacobs et al. 2016). The CMR (Carcinogenicity, Mutagenicity, Reproductive Toxicity) endpoints are thus not typically critical to ensure minimum safety requirements before entering clinical trials. At this stage, in the specific context of COVID-19 (and as mentioned above), drug repurposing is currently the focus. These requirements are usually waived, since treatment is less than 6 months. No data are particularly needed, only a request for full contraception with pregnancy tests every month to cover fertility under clinical trials. What is needed is a repeated dose test, but maximally of one-month length. It would be a great opportunity to complete such a data set with MPS, since some of them do cover up to 28 days and are adapted to dose-repetition. MPS are not “qualified,” however, and therefore difficult to use in a regulatory context (although there are examples of platforms ready to be used for vaccine preclinical testing (Sanchez-Schmitz et al. 2018). The recently published ‘EMA regulatory strategy 2025’ reports, “One reason for hesitancy may be concerns on the part of developers as well as industry that use of such New Approach Methodologies (NAMs) will not be acceptable to regulators and will thus stall approvals. Other possible reasons for the limited use of NAMs include a lack of knowledge regarding the existence or the exact functioning of such models, lack of model validation, or high costs associated with their implementation.” (EMA 2020b).

As mentioned above, since the focus currently is on drug repurposing, most of the submission proposals are rich in safety information already, but considerations for the very ill patient group and the impact of other treatments applied at the same time are critical. As long as there is no standard of care, proper control is difficult to define.

Concerning (3), excellent NAM are available to test drug-drug interactions at the level of a transporter and drug-metabolizing enzymes. This is well-established by drug discovery companies, and therefore such methods allow a quick transition to further testing in humans.

A major question for deciding on test methods (NAM vs*.* animal) is: “Can we prevent harm from trial participants and, in the end, patients?” Olson et al. (2000) showed quite impressively the limits of predicting human adverse effects with animal testing. More recently, (Tamaki et al. 2013) studied 1256 adverse drug reactions after the administration of 142 drugs approved in Japan from 2001 to 2010. Only about half of them were predicted by pre-clinical (animal) data. Even less predictivity was found by Bailey et al. (2014, 2015) for 2366 drugs. Two industry groups, however—Monticello et al. (2017) and Clark and Steger-Hartmann (2018)—had more positive results.

It should be noted that the problem does not end with clinical trials. Of 578 discontinued and withdrawn drugs in Europe and the US, almost half were discontinued due to toxicity (Siramshetty et al. 2016).

## Use of NAM for batch release testing (quality)

In many countries, governmental quality control of human vaccines is a long-established tradition. In the US, on July 1, 1902, the US Congress passed “An act to regulate the sale of viruses, serums, toxins, and analogous products,” later referred to as the Biologics Control Act. This was the first modern federal legislation to control the quality of drugs, in part as a response to 1901 contamination events in St. Louis and Camden involving smallpox vaccine and diphtheria antitoxin (https://bit.ly/3cONhKl). The Act created the Hygienic Laboratory of the US Public Health Service to oversee the manufacture of biological drugs, which eventually became the National Institutes of Health. Vaccine approval in the US is responsibility of the FDA, but also after licensure the agency continues to monitor the production of vaccines, including inspecting facilities and reviewing the manufacturer’s tests of lots of vaccines for potency, safety, and purity. The FDA has the right to conduct testing of manufacturers’ vaccines.

In 1994 in Europe, the so-called Official Control Authority Batch Release Network (OCABR) was implemented, shortly after the establishment of the European Union. Today, Official Medicinal Control Laboratories (OMCLs) are part of the European OCABR Network. In many European countries, OMCLs experimentally test every batch of human vaccines before they enter the market. For influenza, for example, that amounted to about 6000 batches in Europe between 2006 and 2016 (Kretzschmar et al. 2018). This type of batch control testing is among the most animal-consuming in regulatory testing, and especially where actual challenge tests infecting the animals is required, constitutes the most severe suffering inflicted on laboratory animals (Busquet et al. 2020). Major progress for many vaccines has been made converting to serological tests, i.e., where animals’ antibody titers or the protective value of the antibodies induced is measured and no infection of the animals (to show protection) is carried out. Genetically (or otherwise engineered) vaccines developed for COVID-19 may have reduced need for batch release control on animals. Alternatively, serological or non-animal batch release tests may be implemented from the beginning (De Mattia et al. 2011).

## Conclusions

The field of 3Rs (replacement, reduction, refinement) is prolific for the regulation of vaccines and pharmaceutical drugs (Goh et al. 2015; Eskes and Whelan 2016; Sewell et al. 2016), thanks to constant input from regulators, academics, pharmaceutical companies, and EU funding bodies. As stated by the WHO technical report on non-clinical evaluation of vaccines (WHO 2003): “Product diversity and new approaches, technologies, and methodologies develop over time; therefore, judgment based on the best science available should always form the basis for deciding on the type and extent of nonclinical evaluation for these products.” In this context—artificial intelligence, new approach methods (NAM), in vitro*, and *in silico models—high-throughput screening methods can play a crucial role. These technologies and methods have consistently proved to be human-relevant and effective, allowing safe progression to clinical testing in a shorter amount of time as compared to traditional animal testing. Using high-throughput screening methods, for example, could rapidly qualify or disqualify any drugs candidates or facilitate repurposing of approved therapeutics in treating patients with the COVID-19. Over the past 20–30 years, animal-free methods have developed impressively, most notably thanks to EU-funded projects under IMI, and could be effectively employed, especially in predicting toxicity of potential COVID-19 vaccines. Read-across, as developed intensively by ECHA (European Chemical Agency) and under the Horizon 2020-funded EU-ToxRisk (Escher et al. 2019), could also contribute significantly and should, therefore, be fully exploited by EMA and the Member States when information is missing for more than impurities.

In the specific context of the COVID-19 pandemic, EMA has committed to “... *making use of all available tools and platforms to facilitate research and development and subsequent rapid assessment of medicines for COVID-19*” (reference provided in the Supplementary Material). Additionally, the agency has expressed the will to take part in an open discussion on the use of NAM for COVID-19 vaccines and treatment development (reference provided in the Supplementary Material).

On March 18, the first ICMRA (International Coalition of Medicines Regulatory Authorities) “regulatory workshop on COVID-19” took place, with experts from the WHO, the EC, EMA, and FDA. As part of this debate, the experts discussed preclinical data required to support first-in-human (FIH) clinical trials, including which animal studies would *not* be needed prior to proceeding to FIH clinical trials. The summary report indicates that the efficacy of the SARS-CoV-2 vaccine candidate is not required to be demonstrated in animal challenge models prior to proceeding to FIH clinical trials (IMCRA 2020).

For safety testing requirements, the road is more challenging. At the International Council for Harmonisation of Technical Requirements for Pharmaceuticals for Human Use (ICH), NAM are accepted only in exceptional circumstances, as described in the recent update of the S5 guideline on detection of reproductive and developmental toxicity for human pharmaceuticals, which allows the use of NAM for the preclinical phase in a specific regulatory condition (such as life-threatening disease) (ICH 2020). Governmental organizations, such as FDA in the USA or EMA in the EU, have specific programs to implement the use of NAMs in safety and efficacy assessment. The proof-of-concept program is described by EMA in the guideline on quality, non-clinical and clinical requirements for investigational Advanced Therapy Medicinal Products (ATMPs) in clinical trials. Here, the use of in vitro and ex vivo cell and tissue-based models are stated (EMA 2019) to be considered “... to supplement or substitute in vivo animal studies to demonstrate the proof of concept.” This would open a possibility for the use of human tissue, 3D models (MPS, organ-on-chip, spheroid models), and in silico approaches to allow faster development to provide a drug within a year, as called for by several decision-makers and by the global scientific community.

## Electronic supplementary material

Below is the link to the electronic supplementary material.

Supplementary file1 (PDF 573 kb)
