# Supplementary material for: Harnessing the power of novel animal-free test methods for the development of COVID-19 drugs and vaccines
Source: Arch Toxicol. 2020 May 23;94(6):2263–72. doi: 10.1007/s00204-020-02787-2 (PMC7245508; doi:10.1007/s00204-020-02787-2)
Supplement: Supplementary file 1 — Supplementary file1 (PDF 573 kb) [file 204_2020_2787_MOESM1_ESM.pdf]

Supplementary material

Harnessing the power of novel animal-free test methods for the development of COVID-19 drugs and vaccines

Francois Busquet, Thomas Hartung, Giorgia Pallocca, Costanza Rovida, Marcel Leist*

*Marcel Leist, PhD
In vitro Toxicology and Biomedicine, Dept inaugurated by the Doerenkamp-Zbinden Foundation at the University of Konstanz
University of Konstanz
78457 Konstanz/Germany
Tel: +49 (0) 7531 88 5037 (Fax 5039)
Email: marcel.leist@uni-konstanz.de

Description of the material:

- Official response by EMA executive director, Prof. Guido Rasi, to a letter presented by 18 Members of the European Parliament (MEPs) calling for new R&D approaches for COVID-19 vaccines and treatment.

EUROPEAN MEDICINES AGENCY
SCIENCE MEDICINES HEALTH

To the attention of:
Eleonora EVI, MEP
Tilly METZ, MEP
Anja HAZEKAMP, MEP
Francisco GUERREIRO, MEP
Sirpa PIETIKÄINEN, MEP
Sylwia SPUREK, MEP
Michèle RIVASI, MEP
Caroline ROOSE, MEP
Klemen GROSELJ, MEP
Virginie JORON, MEP
Clare DALY, MEP
Attila ARA-KOVACS, MEP
Andrus ANSIP, MEP
Aurélia BEIGNEUX, MEP
Rosa D'AMATO, MEP
Martin BUSCHMANN, MEP
Dino GIARRUSSO, MEP
Juozas OLEKAS, MEP

22 April 2020
EMA/210252/2020
Executive Director

Dear honourable Members of the European Parliament,

Subject: Reply to your letter on new R&D approaches for COVID-19 vaccines and treatments

Thank you for your letter dated 6 April on the need to adopt new approaches and best available technology to rapidly develop COVID-19 vaccines and treatments. I fully agree with you that the spread of the pandemic has become very significant and that there is an urgent need to discover and promptly authorise new treatments and vaccines. With this letter I would like to reassure you that we are devoting our best scientific experts to that end and making use of all available tools and platforms to facilitate research and development and subsequent rapid assessment of medicines for COVID-19.

Firstly, I would like to inform you that on 9 April EMA announced the creation of the COVID-19 EMA pandemic Task Force (COVID-ETF)[1]. This dedicated COVID-ETF has been set-up in line with EMA's Health Threat Plan and brings together the best scientific expertise from the EU medicines regulatory network, dedicated to facilitating and ultimately accelerating the development, authorisation and safety monitoring of therapeutics and vaccines intended for treatment or prevention of COVID-19.

[1] https://www.ema.europa.eu/en/news/ema-establishes-task-force-take-quick-coordinated-regulatory-action-related-covid-19-medicines

Official address Domenico Scarlattilaan 6 ● 1083 HS Amsterdam ● The Netherlands
Address for visits and deliveries Refer to www.ema.europa.eu/how-to-find-us
Send us a question Go to www.ema.europa.eu/contact **Telephone** +31 (0)88 781 6000
An agency of the European Union

© European Medicines Agency, 2020. Reproduction is authorised provided the source is acknowledged.

The COVID-19 ETF will be at the disposal of the European Commission and all Member States to provide EU-wide scientific recommendations on any relevant aspect, including pre-clinical testing and clinical trial design, methodologies for the collection of post-marketing real-world evidence etc.

Secondly, I note your call to adopt "animal-free methods" to predict toxicity of potential COVID-19 vaccines, instead of following traditional R&D approaches. I would like to let you know in this regard that, since 2010, EMA has set up a formal EU expert group[2] on the application of replacement, reduction and refinement (the '3Rs') of animal testing in the development of medicinal products. Through development of harmonised guidance, the 3Rs expert group has made a significant contribution towards the elimination of repetitious and unnecessary animal testing within the EU.
In addition, EMA is available to discuss with companies and validate new methods or technologies to replace animal testing, through its scientific advice pathways[3].
It remains the case, however, that certain types of data can and should only be generated by means of animal studies, before exposing humans to a new medicine.

This brings me to your call to "adopt new approaches and, when feasible, safe straight-to-human vaccine and drug trials in order not to delay the R&D process". I would like to inform you in this regard that the EMA has convened, under the umbrella of the International Coalition of Medicines Regulatory Authorities (ICMRA), which I am currently chairing, all global medicine regulators to map out data requirements for phase 1 COVID-19 vaccine trials. As part of this exercise, during the first ICMRA workshop on COVID-19 vaccine development held on 18 March 2020[4] our experts have been discussing preclinical data required to support proceeding to first-in-human (FIH) clinical trials, including what animal studies would not need to be conducted prior to proceeding to FIH clinical trials.
In addition, via ICMRA and also via EMA's COVID-ETF direct interaction with companies, EMA has advised developers to leverage knowledge accumulated with 'platform technology' to accelerate the development of a SARS-CoV-2 vaccine manufactured building on existing toxicology and clinical data obtained from other medicines using the same platform, without the need to repeat certain studies. More details on such considerations are available in the public report of the 1st ICMRA workshop on COVID-19 vaccine development.

I hope this clarifies our commitment to work in the direction you indicated. As you will surely all agree, in particular as these vaccines will have to be safely administered to millions of people, it will be crucial to ensure the right balance between rapid development of vaccines and the need to generate enough robust data to enable regulatory decision-making for the benefit of public health.

I remain available in case you have any further questions.

Kind regards

Guido Rasi
Executive Director

[2] https://www.ema.europa.eu/en/committees/working-parties-other-groups/chmp/expert-group-3rs.
[3]https://www.ema.europa.eu/en/human-regulatory/research-development/scientific-advice-protocol-assistance/qualification-novel-methodologies-medicine-development.
[4] https://www.ema.europa.eu/en/news/global-regulators-map-out-data-requirements-phase-1-covid-19-vaccine-trials
